# Supplementary material for: Vitamin D Modified DSS-Induced Colitis in Mice via STING Signaling Pathway
Source: Biology (Basel). 2025 Jun 18;14(6):715. doi: 10.3390/biology14060715 (PMC12190092; doi:10.3390/biology14060715)
Supplement: Supplementary file 1 [file biology-14-00715-s001.zip › Supplementary Materials S1: 16s-report of gut microbiota/Groups/FAPROTAX/FAPROTAX_heatmap.pdf]

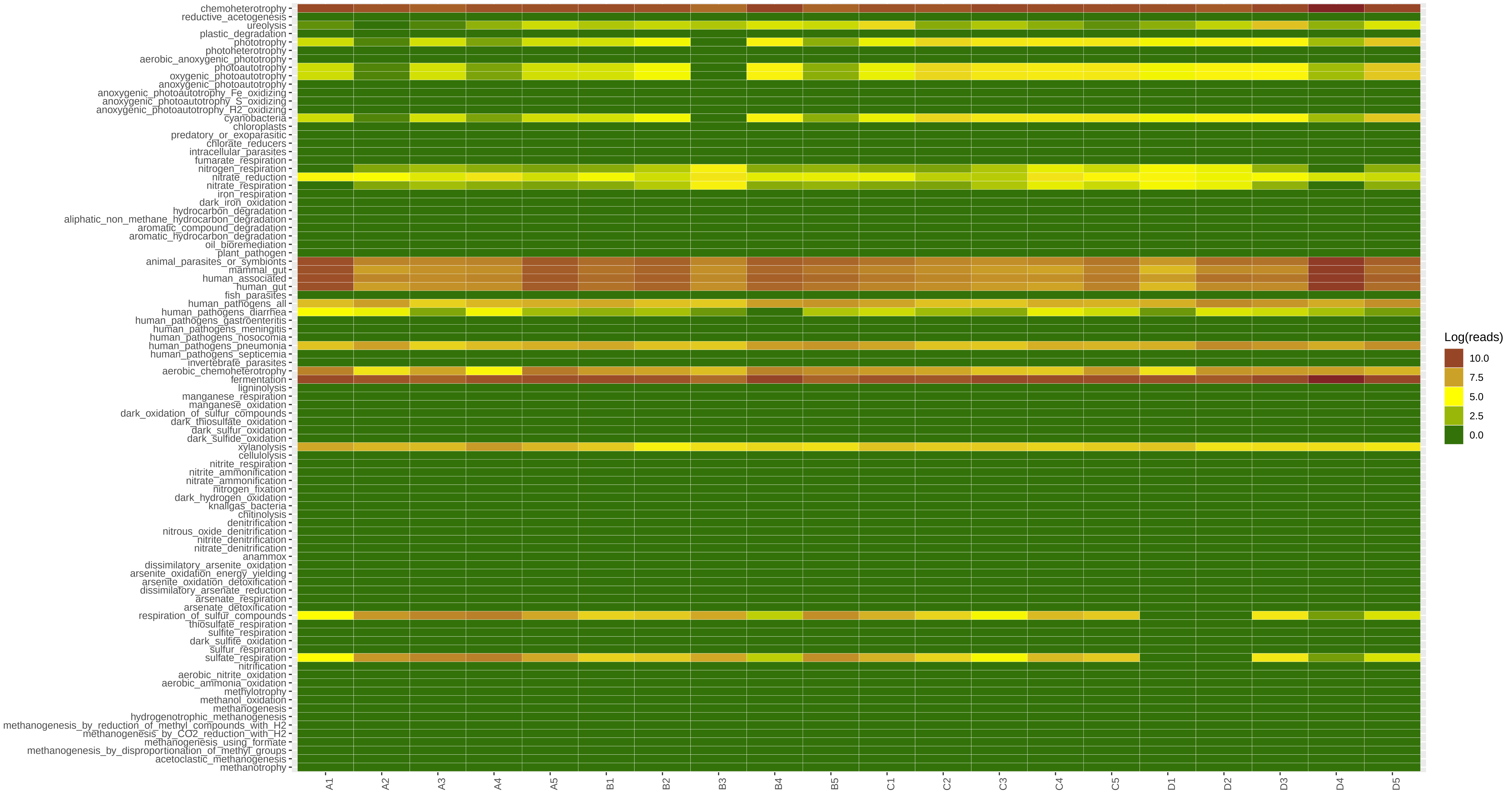

chemoheterotrophy
reductive_acetogenesis
ureolysis
plastic_degradation
phototrophy
photoheterotrophy
aerobic_anoxygenic_phototrophy
photoautotrophy
oxygenic_photoautotrophy
anoxygenic_photoautotrophy
anoxygenic_photoautotrophy_Fe_oxidizing
anoxygenic_photoautotrophy_S_oxidizing
anoxygenic_photoautotrophy_H2_oxidizing
cyanobacteria
chloroplasts
predatory_or_exoparasitic
chlorate_reducers
intracellular_parasites
fumarate_respiration
nitrogen_respiration
nitrate_reduction
nitrate_respiration
iron_respiration
dark_iron_oxidation
hydrocarbon_degradation
aliphatic_non_methane_hydrocarbon_degradation
aromatic_compound_degradation
aromatic_hydrocarbon_degradation
oil_bioremediation
plant_pathogen
animal_parasites_or_symbionts
mammal_gut
human_associated
human_gut
fish_parasites
human_pathogens_all
human_pathogens_diarrhea
human_pathogens_gastroenteritis
human_pathogens_meningitis
human_pathogens_nosocomia
human_pathogens_pneumonia
human_pathogens_septicemia
invertebrate_parasites
aerobic_chemoheterotrophy
fermentation
ligninolysis
manganese_respiration
manganese_oxidation
dark_oxidation_of_sulfur_compounds
dark_thiosulfate_oxidation
dark_sulfur_oxidation
dark_sulfide_oxidation
xylanolysis
cellulolysis
nitrite_respiration
nitrite_ammonification
nitrate_ammonification
nitrogen_fixation
dark_hydrogen_oxidation
knallgas_bacteria
chitinolysis
denitrification
nitrous_oxide_denitrification
nitrite_denitrification
nitrate_denitrification
anammox
dissimilatory_arsenite_oxidation
arsenite_oxidation_energy_yielding
arsenite_oxidation_detoxification
dissimilatory_arsenate_reduction
arsenate_respiration
arsenate_detoxification
respiration_of_sulfur_compounds
thiosulfate_respiration
sulfite_respiration
dark_sulfite_oxidation
sulfur_respiration
sulfate_respiration
nitrification
aerobic_nitrite_oxidation
aerobic_ammonia_oxidation
methylotrophy
methanol_oxidation
methanogenesis
hydrogenotrophic_methanogenesis
methanogenesis_by_reduction_of_methyl_compounds_with_H2
methanogenesis_by_CO2_reduction_with_H2
methanogenesis_using_formate
methanogenesis_by_disproportionation_of_methyl_groups
acetoclastic_methanogenesis
methanotrophy
A1
A2
A3
A4
A5
B1
B2
B3
B4
B5
C1
C2
C3
C4
C5
D1
D2
D3
D4
D5
Log(reads)
10.0
7.5
5.0
2.5
0.0
